# A Case of Pemphigus Herpetiformis in a 12-Year-Old Male

**DOI:** 10.5402/2011/712560

**Published:** 2011-04-07

**Authors:** O. Hocar, I. Ait Sab, N. Akhdari, M. Hakkou, S. Amal

**Affiliations:** ^1^Dermatology Department, School of Medicine, Cadi Ayyad University, Mohammed the VIth University Hospital Marrakesh, Morocco; ^2^Paediatrics Department, School of Medicine, Cadi Ayyad University, Mohammed the VIth University Hospital Marrakesh, Morocco; ^3^Pathology Group laboratory of Marrakesh, Morocco

## Abstract

Pemphigus herpetiformis (PH) is one of the less common forms of pemphigus. PH in children is unreported. We describe a case of a child who developed PH. *Observation*. A 12-year-old boy was seen at our department with erosive plaques, vesicles, and crusted cutaneous lesions associated with severe itching persisting for six months. Histologic examination showed an intraepidermal bulla containing rare acantholytic epidermal cells with eosinophilic spongiosis. Direct immunofluorescence demonstrated intercellular Ig G and C3 deposit. The serum titer of antibodies against intercellular epidermal was 1/200 UI/l. Diagnosis of PH was made, and treatment with Dapsone 2 mg/kg per day resulted in total clinical remission. However, two months later, new vesicles reappeared and treatment was begun with prednisone at a dose of 2 mg/kg daily. There was a very good response. *Discussion*. Childhood pemphigus herpetiformis is a rare disease, often initially misdiagnosed. It must not be forgotten that the disease is a possible cause of erosive mucocutaneous disease in children.

## 1. Introduction

The pemphigus diseases, which include some of the most severe bullous autoimmune skin reactions, are seen predominantly in middle-aged and elderly individuals. Only endemic pemphigus foliaceus in South America most frequently affects juveniles and children. All nonendemic pemphigus diseases, including paraneoplastic pemphigus, have been reported to occur in adolescents and even very rarely in children younger than 10 years.

Pemphigus herpetiformis (PH) is considered a variant of pemphigus displaying clinical features similar to dermatitis herpetiformis and a diverse histopathologic pattern with intraepidermal and subcorneal microabscesses, eosinophilic spongiosis, or superficial bullae with usually scant acantholytic cells [1, 2]. The clinical picture is variable, often with coalescent annular or gyrate vesiculopustular lesions. The diagnosis is based on detection of Ig G antikeratinocyte cell surface antibodies, both bound in vivo and in circulation.

In the literature, few cases of pemphigus are reported in children. This rarity in childhood may be only apparent as a result of its difficult diagnosis. Pemphigus herpetiformis in a child is unreported. We describe a case that developed PH.

## 2. Observation

**Figure 1:**
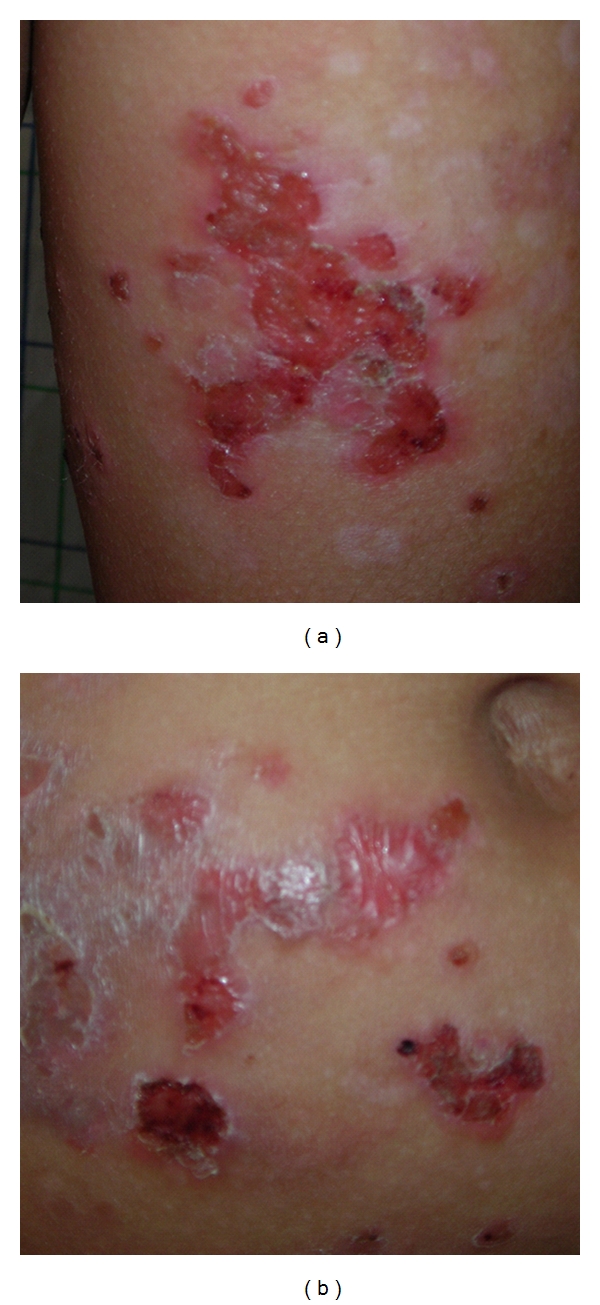
(a) Erosive plaque on the arm in pemphigus herpetiformis. (b) Arciform pattern of postbullous lesion in pemphigus herpetiformis.

**Figure 2:**
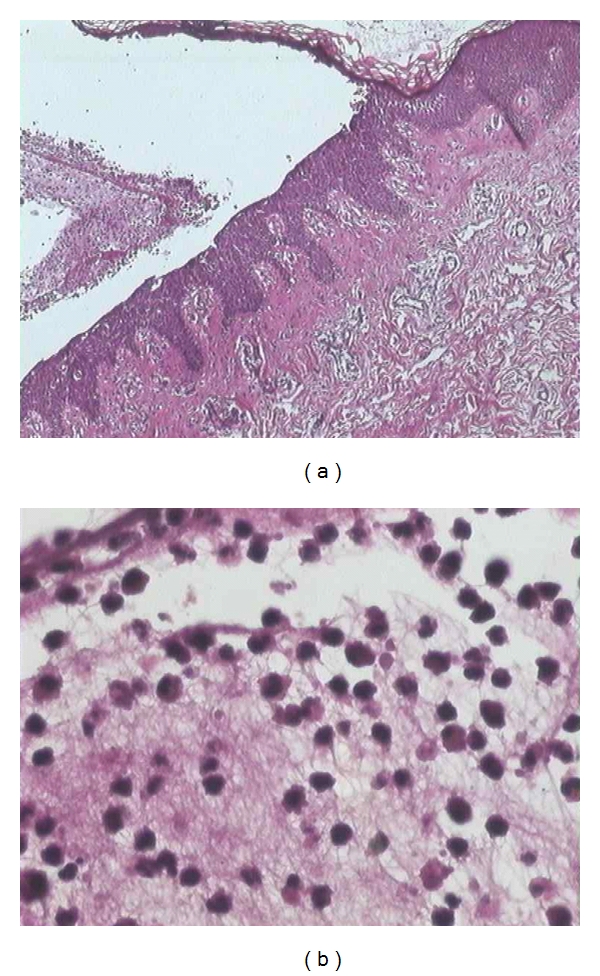
Histopathologic features. Intraepidermal bulla filled with neutrophils and numerous eosinophils. Single acantholytic cells are seen. Lower epidermis: eosinophilic spongiosis (HE ×40) Intraepidermal bulla filled with neutrophils and eosinophils, characteristic of eosinophilic spongiosis (HE ×200)

**Figure 3:**
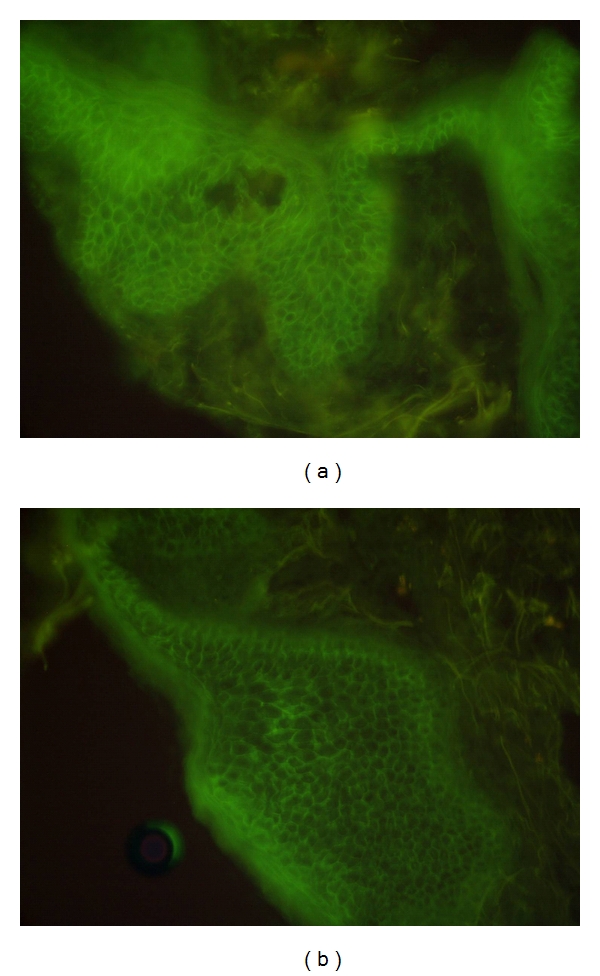
(a, b) Direct immunofluorescence. IgG and C3 cell surface deposits throughout the whole epidermis.

An 12-year-old boy was seen at our department in March, 2007, with erosive plaques, vesicles, bulls, and crusted lesions associated with severe itching persisting for six months. The boy's family history and personal history were unremarkable, and his growth and development had proceeded normally. On examination, we observed vesicular, bullous lesions, some having clear contents and some being purulent with erosive arciform plaques and crusted lesions (Figures 1(a) and 1(b)). They were seen especially over the back, buttocks, chest, abdomen legs, and arms. The face, scalp, palms, and soles were spared. The mucosa and nails were normal. Nikolsky's sign was negative. Results of physical examination were completely normal, except for the cutaneous findings. Laboratory values were within normal limits. Histologic examination of one of the lesions showed an intraepidermal bulla containing rare acantholytic epidermal cells, eosinophil and neurophil cells. The lower epidermis showed eosinophilic spongiosis and focal acanthosis. The superticia1 and reticular dermis had an inflammatory infiltrate of eosinophile and neutrophile around some of the blood vessels (Figures 2(a) and 2(b)). Direct immunofluorescence demonstrated intercellular IgG and complement C3. IgA intercellular deposits on epidermis were not seen (Figures 3(a) and 3(b)). The serum titer of antibodies against intercellular epidermal was positive (1/200 UI/l). Antiendomysial and antigliadin antibodies serum titers were negative. A diagnosis of PH was made, and treatment with Dapsone 2 mg/kg per day resulted in total clinical remission. However, two months later, new vesicles reappeared and treatment was begun with prednisone at a dose of 2 mg/kg daily. There was a very good response; lesions regressed slowly, until they completely disappeared after 4 weeks of treatment. The daily dose of the prednisone was lowered gradually according to the clinical improvement, and, at the time of this report, one year after onset of the disease the skin involvement was being kept under control on a low maintenance dose of 10 mg of prednisone daily.

## 3. Discussion

Autoimmune blistering diseases are extremely rare in children [3]. A review from a paediatric dermatology referral center for a population of 4 million people identified only 23 cases of immunobullous disease in patients less than 18 years of age over a 16-year period [4]. Of all the immunobullous disorders in children, pemphigus comprises a minority. A recent review of pemphigus vulgaris (PV) in children found only 46 cases reported in the literature [5]. Cases with PH are quite uncommon.

Pemphigus herpetiformis (PH) was first introduced by Jablonska and colleagues [1] as a variant of pemphigus. Various terms have been used to describe this condition, such as acantholytic herpetiform dermatitis [6], pemphigus controlled by sulfapyridine [7], and mixed bullous disease [8]. Clinical manifestations consist of erythematous, urticarial plaques, and vesicles that present in herpetiform arrangement [8]. Histological findings of HP are variable and include eosinophilic spongiosis and subcorneal pustules with minimal or no apparent acantholysis [9, 10]. Immunofluorescence findings show that most patients with PH have IgG antibodies against keratinocyte cell surfaces [9]. Dapsone is the first choice of drug in the PH treatment given as monotherapy or combined with systemic corticosteroids [2]. In most reported cases, the use of systemic corticosteroids was necessary to achieve clinical remission [2]. In some cases, however, the use of immunosuppressive drugs such as azathioprine and cyclophosphamide becomes necessary to induce clinical remission or to help in reducing steroid dose so that its collateral effects are minimized [2]. Its position in the pemphigus group remains controversial. Based on its clinical features, some researchers reported the disease as a distinct entity and consider it to be different from classic pemphigus because of its clinical peculiarity and benign course. However, others described it as a variant of pemphigus foliaceus (PF) or PV [9].

We believe the boy presents a pemphigus herpetiformis variant because of the clinical features of dermatitis herpetiformis (vesicles, bulls, and itching) and the immunologic (IgG and C3 deposit on intercellular epidermal) and histological features (intraepidermal bulls and acantholysis) of pemphigus with an eosinophilic spongiosis. 

Various types of pemphigus including vulgaris, foliaceus, erythematosus, vegetans, and paraneoplastic pemphigus can be seen in childhood [10]. We could not find reports of pemphigus herpetiformis in childhood other than our patient. Childhood pemphigus herpetiformis is a rare disease, often initially misdiagnosed. The disease must not be forgotten as a possible cause of erosive mucocutaneous disease in children. It is important that antibody titers, management plans, and outcomes for this disease be reported so that greater knowledge of the effects of various treatment regimens can be obtained.

## Abbreviations

Dsg 3: Desmoglein 3
Dsg 1: Desmoglein 1
PF: Pemphigus foliaceus
PH: Pemphigus herpetiformis
PV: Pemphigus vulgaris.

## References

[1] Jablonska S, Chorzelski TP, Beutner EH, Chorzelska J (1975). Herpetiform pemphigus, a variable pattern of pemphigus. *International Journal of Dermatology*.

[2] Maciejowska E, Jablonska S, Chorzelski T (1987). Is pemphigus herpetiformis an entity?. *International Journal of Dermatology*.

[3] Rabinowitz LG, Esterly NB (1993). Inflammatory bullous diseases in children. *Dermatologic Clinics*.

[4] Weston WL, Morelli JG, Huff JC (1997). Misdiagnosis, treatments, and outcomes in the immunobullous diseases in children. *Pediatric Dermatology*.

[5] Bjarnason B, Flosadóttir E (1999). Childhood, neonatal, and stillborn pemphigus vulgaris. *International Journal of Dermatology*.

[6] DeMento FJ, Grover RW (1973). Acantholytic herpetiform dermatitis. *Archives of Dermatology*.

[7] Seah PP, Fry L, Cairns RJ, Feiwel M (1973). Pemphigus controlled by sulphapyridine. *British Journal of Dermatology*.

[8] Barranco VP (1974). Mixed bullous disease. *Archives of Dermatology*.

[9] Miura T, Kawakami Y, Oyama N (2010). A case of pemphigus herpetiformis with absence of antibodies to desmogleins 1 and 3. *Journal of the European Academy of Dermatology and Venereology*.

[10] Wananukul S, Pongprasit P (1999). Childhood pemphigus. *International Journal of Dermatology*.

